# Powassan Virus Encephalitis in an Immunocompromised Patient: A Diagnostic Challenge With Case Report and Literature Review

**DOI:** 10.1155/crdi/4453384

**Published:** 2025-09-16

**Authors:** Andrew Johnston, Johnathone Yang, Elizabeth Schiffman, Alison Galdys, Lauren Fontana

**Affiliations:** ^1^University of Minnesota Medical School, Minneapolis, Minnesota, USA; ^2^Division of Infectious Diseases, University of Minnesota Medical School, Minneapolis, Minnesota, USA; ^3^Vector Borne Diseases Unit, Minnesota Department of Health and Public Health Lab, St. Paul, Minnesota, USA

**Keywords:** arbovirus infections, immunocompromised, Jamestown Canyon virus, Powassan virus, serology

## Abstract

Powassan virus (POWV) is a tick-borne flavivirus transmitted to humans by *Ixodes* ticks. In this report, we describe an immunocompromised patient who presented with progressive neurologic symptoms and was diagnosed with POWV encephalitis. Additionally, the patient tested positive for Jamestown Canyon virus (JCV), a mosquito-borne *orthobunyavirus*, creating a diagnostic dilemma. This case underscores the importance of considering vector-borne illnesses in immunocompromised individuals who present with neurologic symptoms, particularly during peak arboviral transmission seasons in the United States. It also highlights the complexities of laboratory testing for arboviral infections in these vulnerable patients.

## 1. Introduction

Powassan virus (POWV) is a tick-borne, positive sense RNA virus from the Flaviviridae family first identified in 1958 in Powassan, Ontario. There have been approximately 397 confirmed cases since its discovery, with most cases being identified in the Great Lakes and Northeastern regions of the United States [1]. POWV is maintained in the environment in a cycle that includes small mammals and ticks. Transmission to humans is believed to mostly commonly occur through bites by the *Ixodes scapularis* tick, the primary vector for several pathogens (i.e., *Anaplasma phagocytophilum, Babesia microti,* and *Borrelia burgdorferi*). There are two lineages of POWV, lineage 1 and lineage 2. Lineage 1 is found in tick species that rarely interact with humans, *Ixodes cookei* and *Ixodes marxi*. Majority of all human cases of POWV, except 1 case in 2022, which have undergone molecular characterization, have been attributed to lineage 2 [1–4]. Human cases most commonly occur in a bimodal distribution between April–July and September–October, which aligns with the seasonal distribution patterns of *I. scapularis* [1, 2, 5].

Jamestown Canyon virus (JCV) is a mosquito-borne *orthobunyavirus* first identified in Jamestown Canyon, Colorado in 1961. There have been a total of 336 cases of JCV reported in the United States, and similar to POWV, JCV has a bimodal distribution with cases occurring primarily in the spring and late summer in the Great Lakes and Northeastern regions of the United States [6, 7]. Transmission of JCV commonly occurs through bites from a variety of mosquito species, including those in the *Aedes*, *Culiseta*, and *Coquillettidia genera.* White-tailed deer are thought to be the most common amplifying host of JCV [6, 8].

Epidemiologic investigations have documented an increase in human cases of tick and mosquito borne diseases in the past 10–20 years [9–13]. This upward trend is expected to continue, driven in part by the impacts of climate change [11]. Rising temperatures lead to earlier onset of tick activity in the spring and extend the tick season into the fall, expanding the window for arbovirus transmission [10, 11]. Additionally, milder winter conditions likely improve the survival rates of ticks and their mammalian reservoirs [10]. Cases of POWV and JCV have been reported in the winter months, suggesting that transmission can occur outside the typical season window of spring through fall [13, 14]. A warming climate has the potential to lead to greater population level exposure, expanded geographic involvement, and an increase in the burden of arboviral illnesses.

Symptomatic infections by POWV and JCV frequently begin with a nonspecific febrile illness. While neuroinvasive disease is possible from both infections, severe long-term sequelae and death are more common in POWV infection [2, 3, 15–19]. Here, we describe a case of POWV infection in an immunocompromised patient with severe neuroinvasive disease leading to death and concomitant positive JCV virus serology creating a diagnostic dilemma.

## 2. Case Presentation

In early May, a 64-year-old male with a history of colonic diffuse large B-cell lymphoma (DLBCL) was admitted to a tertiary acute care hospital with 1 week of progressive dysarthria, anomia, generalized weakness, and a fever of 101 °F. Additional medical history included hypertension, hyperlipidemia, obesity, and obstructive sleep apnea. His DLBCL had been diagnosed 6 months earlier, and he completed four cycles of rituximab, cyclophosphamide, doxorubicin hydrochloride, vincristine sulfate, and prednisone (R-CHOP) therapy, with his last dose given 1 month prior to admission. Upon admission, he was considered to be in disease remission.

The patient lived in a suburban area outside a large city, but 2 weeks prior to his admission, he traveled to a recreational property in northern Minnesota. While there he did not recall mosquito or tick bites, but he had spent substantial time outdoors.

On the initial physician exam, the patient was somnolent but could be aroused with verbal or painful stimuli and was oriented only to self. Speech was minimal and dysarthric. Extraocular movements, facial symmetry, and cranial nerve evaluations were normal. Simple commands could be followed, but there was reduced strength in the right arm and normal strength in the left arm and lower extremities. Hyperreflexia was observed in both lower extremities. Cardiac and lung exam were unremarkable. A port was present in the right upper chest.

Complete blood count (CBC) showed a white blood cell count (WBC) of 5.8 × 10e3/μL, hemoglobin of 6.6 g/dL, and platelets of 274 × 10e3/μL. His creatinine was 0.49 mg/dL, alkaline phosphatase (ALP) of 200 IU/L, alanine aminotransferase (ALT) of 22 IU/L, aspartate transaminase (AST) of 26 IU/L, and total bilirubin of 0.2 mg/dL. Admission head computed tomography (CT) and brain magnetic resonance imaging (MRI) did not show evidence of an acute intracranial pathology or meningeal enhancement.

**Figure 1:**
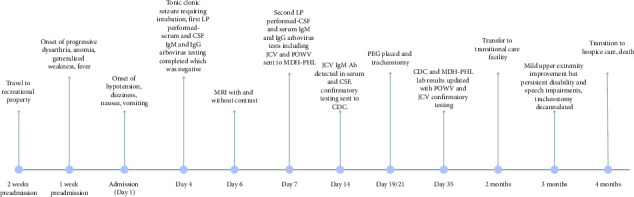
Timeline of events from exposure to death.

**Figure 2:**
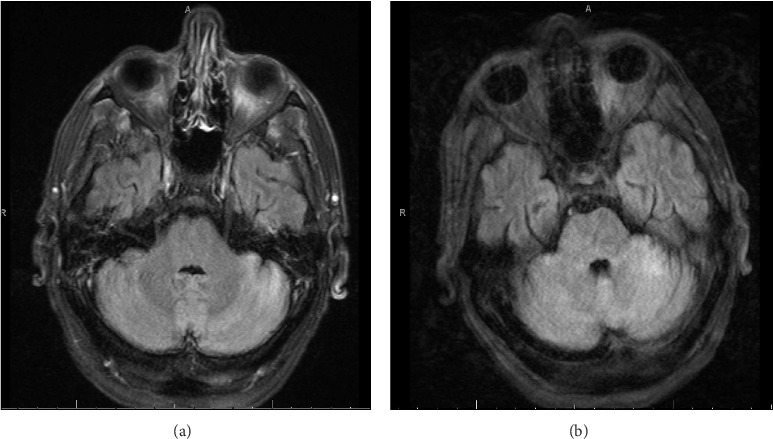
MRI images on day 6 of hospitalization. (a) T2 hyperintense signal involves the superior cerebellum and associated leptomeningeal enhancement. (b) T2 flair sequence.

Figure 1 details the timeline of events. During Day 1 of his admission, he developed hypotension, dizziness, nausea, and vomiting. On Day 4, he experienced a seizure, which required intubation for airway protection. On Days 4 and 7, a lumbar puncture (LP) was performed. The initial LP on day 4 revealed an opening pressure of 21 cm H2O. Cerebrospinal fluid (CSF) studies showed mild pleocytosis with 15 nucleated cells/μL (84% lymphocytes), a protein level of 179 mg/dL, and a borderline hypoglycorrhachia with a glucose level of 48 mg/dL. CSF tests collected on Day 4 were negative for *Cryptococcus* antigen, Herpes simplex virus polymerase chain reaction (PCR), human herpesvirus 6 PCR, varicella zoster virus PCR, human polyomavirus 2 PCR, *Borrelia burgdorferi* IgG/IgM, adenovirus PCR, lymphocytic choriomeningitis virus IgM and IgG, and an autoimmune/paraneoplastic panel including N-methyl-D-aspartate receptor antibody (NMDA) which was completed at Mayo Clinic laboratories. Also, on Day 4, IgM and IgG CSF and serum serologic tests for West Nile virus, St. Louis encephalitis virus, Western equine encephalitis (WEE) virus, Eastern equine encephalitis (EEE) virus, and California virus were negative. On Day 7 serum and, CSF were sent to the Minnesota Department of Health Public Health Laboratory (MDH-PHL) for additional arboviral serologic testing. Again, IgM CSF and serum tests were negative for West Nile virus, St. Louis encephalitis virus, WEE virus, and EEE virus. Additionally, POWV IgM was negative in the serum and CSF. However, IgM antibodies by enzyme immunoassay (EIA) to JCV were detected in CSF and serum (Table 1). The samples were then sent to the Center for Disease Control (CDC) for confirmatory testing. MRI of his brain with and without contrast on day 6 revealed T2 hyperintense signal of the superior cerebellum with leptomeningeal enhancement (Figure 2).

On admission, the patient was started on broad-spectrum antibiotics, including cefepime and vancomycin, which were discontinued on Day 10. On Day 7, intravenous immunoglobulin (IVIG) (0.5 g/kg) was administered for a low serum IgG level of 365 mg/dL. Between hospital Days 10 and 14, the patient received a methylprednisolone burst at 1 g per day, followed by a prednisone taper that was complete one month after admission. During the hospitalization, the patient's alertness, ability to follow commands, dysarthria, and the motor function of the upper and lower extremities worsened. On Days 19 and 21, the patient underwent percutaneous endoscopic gastrostomy (PEG) placement and tracheostomy, respectively.

On Day 35, the patient's physicians were notified of updated results from the CDC and MDH-PHL. CDC testing detected JCV IgM and neutralizing antibodies from the patient's serum, with a PRNT titer of 1:40. Real-time flavivirus PCR (RT-PCR) testing by both CDC and MDH-PHL detected the presence of POWV in the serum and CSF. This test does not specify lineage I or II, but it is presumed to be lineage II since the majority human POWV cases that have undergone molecular characterization have been attributed to lineage II. JCV was not detected by molecular testing (Table 1). The patient remained on supportive care and was discharged to a transitional care facility 2 months after admission.

One month after discharge, the patient showed mild improvement in his ability to move his upper extremities but continued to have significant disabilities and speech difficulties. The tracheostomy tube was decannulated 1 month after discharge. Two months after discharge, he developed failure to thrive and continued to have minimal movement in his lower extremities. The patient required assistance for most movement and activities of daily living. Given the ongoing lack of neurologic improvement and decreased quality of life, the patient was transitioned into hospice care, and he ultimately passed away 4 months after presenting due to the long-term neurological sequelae of his infection.

## 3. Discussion

We present a case of an immunocompromised patient whose clinical presentation and test results were most consistent with a neuroinvasive POWV infection but were complicated by a positive testing for JCV. This case highlights the critical role of careful laboratory testing in diagnosing neuroinvasive arboviral disease in immunocompromised individuals.

### 3.1. Epidemiology

Although POWV and JCV incidences have gradually increased over time, the low prevalence of laboratory-confirmed cases of POWV and JCV, combined with disproportionately high seropositivity rates in certain communities, suggests these infections are underdiagnosed and that current reports underestimate the burden of disease [9, 20–24]. Nationally, 397 *Powassan virus* cases have been reported between 2004 and 2024, with 79 (20%) of these cases occurring in Minnesota (Table 2) [1]. Notably, all of the cases in Minnesota and 94% of the cases nationwide were classified as neuroinvasive infections [1]. The US case fatality rate for POWV is approximately 10%–13%, and 50% of the survivors experience long lasting neurological sequelae [1]. In comparison, the CDC has identified 336 cases of JCV since 2011, with 67% classified as neuroinvasive disease [7]. Comparatively, the US case fatality rate for JCV virus is lower at 3.6% [7]. This data supports the notion that POWV is more commonly associated with neuroinvasive illness and has a higher mortality rate. However, reported cases likely represent the most severe infections, underestimating the true prevalence and overestimating the rates of neuroinvasive illness. Factors contributing to underdiagnosis include the nonspecific nature of disease presentation, diagnostic challenges, patient-specific factors such as the degree of immunosuppression, and limited provider awareness of available testing options [2, 25–27].

### 3.2. Clinical Presentation, Treatment, and Outcomes

As described in Table 2, both JCV and POWV can present as a nonspecific mild febrile illness. However, diagnostic testing is not commonly performed for mild disease and those with benign clinical courses, so it is challenging to know how common these mild cases occur. In more severe and atypical JCV cases, symptoms may progress to meningoencephalitis and have been associated with dysphagia, confusion, seizures, and stroke like illness [13, 28]. The majority of published case reports on JCV neuroinvasive illness, including among those who are immunocompromised, describe patients making full recovery at follow-up or having only minor neurologic sequelae such as fatigue, cognitive difficulties, or expressive aphasia [28–33]. We identified one fatal case of JCV encephalitis in a patient who had received rituximab for mantle cell lymphoma. This case describes rapidly progressive dementia which is not commonly described in the literature for JCV [34].

The patient's long-term neurologic sequelae, arboviral laboratory results, and unfortunate death are most consistent with POWV encephalitis [3, 15–17, 19, 35]. POWV typically causes more severe neuroinvasive disease than JCV with a higher likelihood of progression to meningoencephalitis (Table 2). Severe clinical symptoms include aphasia, dysphagia, dysarthria, cranial nerve abnormalities, and paresis [17, 18, 26, 35]. Prior case reports and series have described worse outcomes in patients with POWV who demonstrate brainstem and cerebellar involvement on MRI [2, 3, 14, 36, 37]. In this case, the admission MRI brain was unremarkable; however, on Day 6 of admission, the MRI revealed a T2 hyperintense signal of the superior cerebellum with leptomeningeal enhancement. The discrepancy between the MRIs on Day 1 and Day 6 is not clear, but it may reflect a delayed radiographic response due to early timing of the initial MRI or a delayed inflammatory response due to the patient's residual immunosuppression after chemotherapy.

Treatment for most of the tick or mosquito borne viruses, including POWV, is primarily supportive, as there are no approved antivirals targeting this infection. It is important to consider the possibility of a coinfection when determining treatment strategies. It is insufficient evidence that patients with a coinfection have more severe illness or a higher mortality rate. For patients not responding to standard treatment, the use of IVIG has been suggested [38]. Cases reports have described favorable responses with immunosuppressive therapy and/or IVIG, suggesting the immune system may play a role to the pathogenesis of illness [36–38]. However, in this case, the patient did not experience a long benefit from IVIG and/or steroids. The use of immunosuppressive therapies and IVIG for treatments remains experimental and requires further investigation.

### 3.3. Arboviral Serologic Testing

Serologic testing is widely available for most endemic and travel associated viruses, so serologic testing is a mainstay for arbovirus testing [25]. For symptomatic cases, the recommendation is to test for arboviral infections initially via IgM serology, followed by confirmatory testing by plaque reduction neutralization testing (PRNT) [25]. Testing for these viruses is not widely available commercially, so most of this is done at state public health labs and the CDC [25]. Molecular methods such as RT-PCR may also be available in specialized labs.

Table 3 outlines the advantages and limitations with arbovirus testing. IgM serology is commonly used in arbovirus testing due to the quick development of IgM antibodies, which can reliably detect seropositivity up to 12 weeks after the infection but can stay positive for much longer [25, 27]. In this case, JCV IgM seropositivity alone could not be diagnostic for acute JCV due to the occurrence of prolonged IgM seropositivity. It is estimated that the baseline seroprevalence for JCV IgM and neutralizing antibodies in endemic areas is approximately 10%–17% among blood donors [24]. Higher rates of IgM seroprevalence among asymptomatic persons suggest that the JCV IgM persists well beyond the acute phase of infection [24]. Compared to IgM testing, IgG serology has few advantages and has a higher risk of cross-reactivity. PRNT measures titers of neutralizing antibodies for a virus and is performed on serum or CSF. However, these tests have a slow turnaround time and are often only available in specialty labs which can result in a delayed diagnosis [25]. Further, the JCV PRNT titer of 1:40 in this case is considered a weak positive and likely did not represent an acute infection. The positive IgM and PRNT in the case we present most likely represented a previous, subclinical JCV infection in which the viremia cleared.

As a part of routine arboviral testing, the MDH-PHL performs sequencing for flaviviruses and bunyaviruses. The PHL does not perform routine sequencing of the Alphaviruses, such as EEE or WEE, but does perform IgM serology. Sequencing of serum and CSF from our patient detected POWV, which CDC later confirmed on serum with RT-PCR. Molecular methods like PCR testing or sequencing are the gold standard for the speciation of most arboviruses but are also more expensive, and testing is restricted to specialty labs [25]. However, molecular methods remain the best option for immunocompromised patients since these patients may test negative on serology due to insufficient or absent antibody production against pathogens. These tests often have a high sensitivity and specificity, though their temporality for utility is often more restrained than other methods. It is not clear how long a person may be viremic with POWV but animal models suggest a detectable viral RNA up to 21 days post infection in the brain [39, 40]. Given the potential for a short viremic period, many patients will present to care too late for accurate testing. Advanced diagnostic lab results were crucial in determining whether the infection was caused by JCV, POWV, or a dual infection.

### 3.4. Impacts of Arboviral Testing in Immunocompromised Patients

The negative *Powassan virus* IgM in this patient was likely due to impaired antibody production and response, which may be attributed to recent rituximab. Rituximab is a monoclonal antibody that targets CD20 cells which are preferentially expressed on the surface of B lymphocytes. Rituximab is included in the standard of care for treatment of DLBCL [41]. It creates antibody-dependent cell-mediated cytotoxicity, thus leading to low CD20 count, depletion of B-cells, and decreased humoral immunity [26, 42]. B cell depletion occurs within a few days of administration and may last for 6–12 months [26]. Importantly, monoclonal antibodies like rituximab selectively target circulating B-cells but not the mature plasma cells in the bone marrow and lymphoid tissue. This helps to explain the preservation of humoral immunity against JCV in this case and his inability to create new antibodies in response to a more recent POWV infection. A 2022 study found that patients on rituximab with severe neuroinvasive disease from an arboviral infection required molecular testing, such as RT-PCR, to make a diagnosis in 20 out of 21 (95%) of patients due to absent antibody response. This study also found a case fatality rate of 79% and those who survived had long-term neurologic sequelae such as cognitive and motor dysfunction [26]. In this case, the patient was unable to mount a sufficient immune response to POWV, which likely contributed to the severity of his illness and the negative POWV IgM.

## 4. Conclusion

This case highlights the importance of considering POWV in immunocompromised patients with febrile encephalopathy, while also recognizing the limitations of arbovirus testing and how patient-specific factors, particularly immunocompromised patients, can influence results. Further research must also be done on the increasing burden of arboviral illnesses in the setting of a warming climate. Enhancing provider education on arboviral testing, fostering collaboration with public health officials, and expanding diagnostic capabilities will be essential in monitoring and addressing the impact of arbovirus infections.

## Figures and Tables

**Table 1:** Powassan and JCV patient testing results.

Sample type	Test method	Powassan virus^1^	Jamestown Canyon virus^1^
Serum	IgM	Negative	Positive
	PRNT	Not done	1:40
	Molecular RT-PCR	Positive	Negative

CSF	IgM	Negative	Positive
	PRNT	Not done	Not done
	Molecular RT-PCR	Positive	Negative

Urine	Molecular	Negative	Not performed

Abbreviations: CSF = cerebral spinal fluid, PRNT = plaque reduction neutralization testing, RT-PCR = reverse transcriptase polymerase chain reaction.

^1^Testing performed at Minnesota department of health and public health laboratory and/or CDC.

**Table 2:** Clinical and epidemiological comparison between Powassan virus and Jamestown Canyon virus.

Name of virus	Powassan virus	Jamestown Canyon virus
Vector	Tick-borne, mostly Ixodes scapularis	Mosquito-borne, including Aedes, Culesita, Coquillettidia spp

Region	Great Lakes and Northeast region of the United States	Great Lakes and Northeast region of the United States

Virus family	Flaviviridae	Peribunyaviridae

Case timing	Bimodal distribution, spring and late summer	Bimodal distribution, spring and late summer

Number of reported cases	397 cases since 2004, 374 identified as neuroinvasive (94%)	336 cases since 2011, 227 identified as neuroinvasive (67%)

Case fatality rate	10%–13%	3.6%

Clinical characteristics	• Nonspecific mild febrile illness	• Asymptomatic or nonspecific mild febrile illness
	• May progress to neuroinvasive illness with meningitis or encephalitis	• Rarely progresses to neuroinvasive illness with meningitis or encephalitis
	• Quickly leads to dysphagia, dysarthria, seizures, vomiting, dysmetria, paralysis	• Neurological symptoms may progress to speech difficulties, confusion, seizures

Long-term sequelae	• Long-term neurological sequelae are common (∼50% of confirmed cases)	• Long-term sequelae are less common
	• Severe neurologic defects such as ataxia, expressive aphasia, cognitive difficulties, extremity spasticity, and/or decreased mobility and strength	• If present, more commonly involves fatigue, cognitive difficulties, and/or expressive aphasia

**Table 3:** Advantage and disadvantages of arbovirus diagnostics.

	Advantages	Disadvantages	Rating
IgM serology	• Antibodies develop quickly	• Antibodies may persist > 1 year	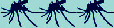
	• Reliable detection up to 12 weeks	• Cross reactivity	

IgG serology	None!	• No information on timing of infection	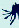
		• Cross reactivity	

Molecular	• Able to speciate	• Very short viremic period	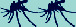
	• Best option for immunocompromised patients	• Typically low viral loads in humans	

PRNT	• Often able to differentiate between related viruses	• Slow turnaround time	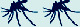
	• Quantifiable titer	• Only available at specialty labs	

*Note:* 
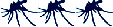
 = best overall choice, 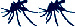 = good, but better with add'l testing, 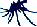 = not recommended for routine dx.

Abbreviation: PRNT = plaque reduction neutralization testing.

## Data Availability

The data that support the findings of this study are available on request from the corresponding author. The data are not publicly available due to privacy or ethical restrictions.
